# Homeobox Transcription Factors Are Required for Fungal Development and the Suppression of Host Defense Mechanisms in the *Colletotrichum scovillei*-Pepper Pathosystem

**DOI:** 10.1128/mBio.01620-21

**Published:** 2021-08-24

**Authors:** Teng Fu, Joon-Hee Han, Jong-Hwan Shin, Hyeunjeong Song, Jaeho Ko, Yong-Hwan Lee, Ki-Tae Kim, Kyoung Su Kim

**Affiliations:** a Division of Bio-Resource Sciences, BioHerb Research Institute, and Interdisciplinary Program in Smart Agriculture, Kangwon National University, Chuncheon, South Korea; b Department of Research and Development, Chuncheon Bioindustry Foundation, Chuncheon, South Korea; c Department of Agricultural Biotechnology, Interdisciplinary Program in Agricultural Genomics, Center for Fungal Genetic Resources, Plant Immunity Research Center, and Research Institute of Agriculture and Life Sciences, Seoul National University, Seoul, South Korea; d Department of Agricultural Life Science, Sunchon National University, Suncheon, South Korea; Cornell University

**Keywords:** anthracnose, appressorium development, *Colletotrichum scovillei*, conidiation, host defense, pathogenicity factor

## Abstract

*Colletotrichum scovillei*, an ascomycete phytopathogenic fungus, is the main causal agent of serious yield losses of economic crops worldwide. The fungus causes anthracnose disease on several fruits, including peppers. However, little is known regarding the underlying molecular mechanisms involved in the development of anthracnose caused by this fungus. In an initial step toward understanding the development of anthracnose on pepper fruits, we retrieved 624 transcription factors (TFs) from the whole genome of *C*. *scovillei* and comparatively analyzed the entire repertoire of TFs among phytopathogenic fungi. Evolution and proliferation of members of the homeobox-like superfamily, including homeobox (HOX) TFs that regulate the development of eukaryotic organisms, were demonstrated in the genus *Colletotrichum*. *C*. *scovillei* was found to contain 10 HOX TF genes (*CsHOX1* to *CsHOX10*), which were functionally characterized using deletion mutants of each *CsHOX* gene. Notably, *CsHOX1* was identified as a pathogenicity factor required for the suppression of host defense mechanisms, which represents a new role for HOX TFs in pathogenic fungi. *CsHOX2* and *CsHOX7* were found to play essential roles in conidiation and appressorium development, respectively, in a stage-specific manner in *C*. *scovillei*. Our study provides a molecular basis for understanding the mechanisms associated with the development of anthracnose on fruits caused by *C*. *scovillei*, which will aid in the development of novel approaches for disease management.

## INTRODUCTION

Pepper (*Capsicum annuum* L.) is an important vegetable crop cultivated in nearly all countries worldwide (1). The yearly worldwide pepper production has been estimated at approximately 36.8 million tons (2). Peppers have been widely used as a food flavoring agent and as medicine for thousands of years. Peppers are good sources of vitamins and minerals; they contain multiple chemical constituents with pharmaceutical properties (1). A major hindrance in pepper production is anthracnose disease caused by *Colletotrichum* species, which are ascomycete fungal pathogens. Anthracnose refers to diseases with the typical symptoms of sunken spots or lesions on infected plant tissues (3, 4). The disease causes substantial economic losses by reducing the productivity and quality of fruits in affected plants (5). Several species in the genus *Colletotrichum* are known to cause anthracnose in pepper plants (6–8). Among them, *C*. *scovillei*, which belongs to the *Colletotrichum acutatum* species complex, is considered a dominant pathogen responsible for serious pepper yield losses in countries in tropical and temperate zones, including Brazil, China, Indonesia, Japan, South Korea, Malaysia, and Thailand (9–15). *C*. *scovillei* has also been reported to attack other economically important crops, such as mango and banana (16, 17). Although most anthracnose disease on fruits can cause considerable losses in yield and quality, the molecular mechanism underlying the development of fruit anthracnose has not yet been elucidated, whereas many foliar diseases in a wide range of plants caused by diverse fungal pathogens have been extensively studied at the molecular level (18–24). Therefore, we have initiated a molecular pathosystem study of *C*. *scovillei* and pepper fruits to better understand anthracnose disease in fruit.

*C. scovillei* produces a large number of conidia that serve as a major inoculum. During the disease cycle, conidia adhere to the surfaces of pepper fruits upon hydration; they then produce germ tubes (7, 25). Appressorium, a specialized infection structure, is differentiated at the tip of the germ tube following the recognition of chemical and physical host signals (26). Considering that *C*. *scovillei* infects only the fruit, the signals for appressorium development on fruit are different from the signals for appressorium development on other tissues of host plants. Similar to several *Colletotrichum* species, *C*. *scovillei* is a hemibiotroph, which exhibits an early biotrophic phase and a late necrotrophic phase during host-pathogen interactions (6, 26–28). At an early stage of appressorium-mediated penetration of *C*. *scovillei*, a unique feature known as a dendroid structure develops in the cuticle layer of pepper fruit; this does not occur in foliar infections involving other *Colletotrichum* pathogens (4, 29, 30). After colonization of host epidermal cells, the fungus develops the typical sunken anthracnose lesion with mucilaginous acervuli containing a large amount of pinkish conidia, which is important for further infection. Polycyclic infection of the fungus contributes to substantial disease during the growing season, thereby causing serious economic losses. Based on the socioeconomic impact and difficulty involved in disease management of fruit anthracnose in a wide range of crops, we previously elucidate the whole genome of *C*. *scovillei* to better understand the molecular mechanisms involved in fruit anthracnose, which have been relatively uncharacterized at the molecular level (8, 31, 32).

Eukaryotic organisms, including fungi, have evolved adaptive genetic responses to a variety of stimuli (33, 34). Transcription factor (TF)-mediated regulation of gene expression plays an important role in controlling crucial aspects of organism survival and development. Gene expression is elaborately orchestrated in a coordinated system of transcription-mediated signaling, in which TFs bind specific sequence elements to initiate or block transcription (35–37). Various TF families are defined by unique DNA-binding motifs (38–40). Although the DNA-binding motifs of TF families are highly conserved across taxa, the members of a particular TF family often have different roles in an organism because of evolutionary divergence (41–43). The availability of genome sequence data has resulted in the identification of a large number of TFs through cross-species comparison, thus enabling TF family-based characterization in functional genomics (44). The homeobox TF family is a prominent TF family associated with fungal development and pathogenicity. Members of this family contain a conserved 60-amino-acid DNA-binding motif known as the homeodomain (45, 46). Since the functional discovery of the homeobox TF family in Drosophila melanogaster, which is expressed in an organ-specific manner during development (47, 48), important roles of homeobox TF orthologs have been established in several fungi (33, 49–52). For example, the rice blast fungal pathogen Magnaporthe oryzae contains seven homeobox TFs, among which *MoHOX2* and *MoHOX7* have been identified as stage-specific key regulators of conidiation and appressorium development, respectively (33, 53). Functional roles of homeobox TFs in infection-related development have been demonstrated in *Colletotrichum orbiculare*, a cucumber anthracnose pathogen, which is evolutionarily very distant from *C*. *scovillei* (54, 55). Unlike *MoHOX7*, the homolog *CoHox3* was associated with maturation of appressoria in *C*. *orbiculare. CoHOX1* was found to be required for pathogenic development, whereas its homolog *MoHOX5* was dispensable for pathogenicity. These results reflect the functional divergence of homeobox TF family members, which further increases the genetic complexities of fungal pathogens with different lifestyles, in response to a wide variety of environmental signals. However, the HOX TF family in pepper fruit anthracnose *C*. *scovillei* has not yet been studied.

To systematically study the functions of *HOX* genes, we first analyzed whole-genome sequences of *C*. *scovillei* and isolated 624 putative TFs (based on InterPro annotation), which contain 48 distinct domains. Through detection of the homeobox domain (IPR001356), a total of 10 HOX TFs (CsHOX1 to CsHOX10) were then obtained from putative TFs in *C*. *scovillei*. These HOX TFs in the homeobox-like domain superfamily (IPR009057) were evolutionarily analyzed in the *Colletotrichum* genus and outgroup species to reveal their origins. To further study the functional roles of *CsHOX* genes in *C*. *scovillei*, we then generated deletion mutants for each *CsHOX* gene, based on homology-dependent gene replacement. Comparative functional analysis of deletion mutants demonstrated that *CsHOX* genes are associated with stage-specific regulations for disease dissemination and development in *C*. *scovillei*. Specifically, *CsHOX2*, *CsHOX7*, and *CsHOX1* were found to be essential for conidiation, appressorium formation, and suppression of the host defense mechanism for anthracnose development on pepper fruits in the *C*. *scovillei*-host pathosystem, respectively. Our findings provide a useful framework for understanding the development of anthracnose disease on fruits caused by *Colletotrichum* species.

## RESULTS

### Distribution of TF and homeobox-related proteins in fungal species including *Colletotrichum* spp.

Our phylogenomic species tree of *Colletotrichum* was consistent with previous phylogenetic trees (56, 57) (see Fig. S1A and Table S1A in the supplemental material). The *C*. *acutatum* species complex diverged from *Colletotrichum orchidophilum*, as previously reported (56). The total number of TFs in *Colletotrichum* species ranged from 337 in *Colletotrichum chlorophyti* to 624 in *C*. *scovillei* (see Fig. S1A and Table S1B and C). The average number of TFs in this genus was 437.0, which was higher than the average number of TFs in the sister taxon *Verticillium* (316.5) but considerably less than the average number of TFs in the distantly related taxon Fusarium (985.7). Among the TFs, the average number of genes encoding a protein with a homeobox-like domain was 46.3 in all selected species (see Table S1D). However, *C*. *scovillei* in the *Colletotrichum* genus and outgroup species, including Fusarium *verticillioides*, Fusarium oxysporum, and Sclerotinia sclerotiorum, had significantly higher numbers of genes than average at 206, 77, 73, and 126 genes, respectively. In contrast, the number of *HOX* genes in the *Colletotrichum* genus consistently ranged from 8 to 11, whereas the number in the outgroup species exhibited considerable variability, from 2 in *Saccharomyces pombe* to 27 in F. verticillioides (see Table S1E). These results indicated that plant pathogens possess more TFs and *HOX* genes than saprotrophs.

10.1128/mBio.01620-21.1 FIG S1 Distributions of TFomes in *Colletotrichum* and related species and phylogenetic tree of proliferated subclade genes and distribution of homeodomain superfamily genes in *Colletotrichum scovillei*. (A) Maximum-likelihood phylogenomic tree, total number of TFs, number of genes belonging to the homeodomain superfamily, and number of *HOX* genes in the selected fungal species are depicted. Average values of each parameter are shown with dashed lines. Lifestyles of fungi and their host types are color coded. Host type is derived from a previous study. Asterisks on the tree represent the genus *Colletotrichum* (**) and the *Colletotrichum acutatum* species complex (*). (B) Distribution of *HOX* genes with their schematic protein structures in the *Colletotrichum* species. Each InterPro domain is color coded and is not to scale. (C) Nodes in the species tree are annotated with the number of genes, number of genes gained (+red), and number of genes lost (–blue). Numbers of genes in leaf nodes represent current numbers of genes that belong to the homeodomain superfamily, including *HOX* genes. Asterisks on the tree represent the genus *Colletotrichum* (**) and *Colletotrichum acutatum* species complex (*). (D) Unrooted phylogenetic tree constructed using homeobox-like domain sequences by the maximum likelihood method and 1000 bootstrap samples. Numbers 1 to 15 represent clades. Red, cyan, and gray circles represent *Colletotrichum scovillei*, other *Colletotrichum* species, and outgroup species, respectively. Genes encoding a protein with a homeobox and a homeobox-like domain are depicted with closed and open circles, respectively. Red arrows represent two *HOX* gene subclades; blue arrow represents a subclade with proliferated homeobox-like domains in *C*. *scovillei*. (E and F) Trees represent *HOX* gene subclades from clade 10 and clade 15, respectively. Download FIG S1, TIF file, 2.7 MB. Copyright © 2021 Fu et al. 2021 Fu et al. https://creativecommons.org/licenses/by/4.0/ This content is distributed under the terms of the Creative Commons Attribution 4.0 International license.

10.1128/mBio.01620-21.7 TABLE S1 (A) List of 30 Fungal species used in this study. (B) List of domains contained by TFs of the selected fungal species. (C) List of TFs in the selected fungal species. (D) List of homeobox-like domain containing genes in the selected fungal species. (E) List of homeobox genes in the selected fungal species. (F) List of 104 different types of domain structures observed in proteins with homeobox-like domains in the selected fungal species. (G) List of genes in each phylogenetic clade of the homeodomain superfamily. Download Table S1, XLSX file, 0.2 MB. Copyright © 2021 Fu et al. 2021 Fu et al. https://creativecommons.org/licenses/by/4.0/ This content is distributed under the terms of the Creative Commons Attribution 4.0 International license.

In total, 104 different types of structures were observed in proteins with homeobox-like domains in the selected fungal species (see Table S1F). Among them, 13 types were proteins with a homeobox domain, although only eight types were detected in *Colletotrichum* species (see Fig. S1B). Within *Colletotrichum* species, proteins that only contained a homeobox domain were most frequently detected. The next most commonly detected HOX proteins had at least two or three C_2_H_2_-type zinc finger domains (IPR007087). A HOX protein with rhodanese-like domain (IPR036873) was present in all members of the *C*. *acutatum* species complex, but sparsely present in other *Colletotrichum* species. Some HOX proteins with C_2_H_2_-type zinc finger domains also had an HTH CenpB-type DNA-binding domain (IPR006600), and were also present in all *Colletotrichum* species, except *Colletotrichum tanaceti*. *C*. *orbiculare* contained one of these proteins, with an additional DUF3425 domain (IPR021833) at the C-terminal end. A HOX protein with three C_2_H_2_-type zinc finger domains, NAD(P)-binding domain (IPR036291), and 6-phosphogluconate dehydrogenase-like C-terminal domain (IPR008927) was only present in *Colletotrichum salicis*.

### Evolution and proliferation of the homeodomain superfamily and *HOX* genes in fungal species, including *Colletotrichum* spp.

Gain and loss analysis of genes encoding members of the homeodomain superfamily, including the *HOX* genes, showed that 35 ancestral genes existed in the last common ancestor of the *Colletotrichum* species complex (see Fig. S1C). The last common ancestor of the *C*. *acutatum* species complex had 37 genes, which remain in *Colletotrichum simmondsii*, *C*. *salicis*, and *C*. *orchidophilum*, while 1 and 2 genes were lost in *Colletotrichum nymphaeae* and *Colletotrichum fioriniae*, respectively. Overall, the phylogenetic tree of the homeodomain superfamily showed that the superfamily of the selected fungal species was divided into 17 clades (see Fig. S1D and Table S1G). The largest clade had 809 genes (clade 15), while the second-largest clade had 211 genes (clade 10); both contained proteins with homeobox domains. Clades 10 and 15 had 148 and 153 *HOX* genes, respectively, which formed a subclade within each clade (see Fig. S1D). The subclades of clades 10 and 15 were further divided into 14 and six families, respectively (see Fig. S1E and S1F). Each subclade contained five *HOX* genes of *C*. *scovillei*. Although subclade 15 only consisted of *HOX* genes, subclade 10 contained a highly diverged homeobox-like domain family (family 3). A pair of *HOX* genes from *Colletotrichum tofieldiae* (KZL74285.1) and *Colletotrichum incanum* (OHW97559.1) (family 2), and a pair of *HOX* genes from *Colletotrichum gloeosporioides* (EQB57487.1) and *Colletotrichum fructicola* (ELA29112.1) (family 10), each independently formed their own families (see Fig. S1E).

Another notable feature of the tree was that the genes encoding proteins with homeobox-like domains in *C*. *scovillei* and *S*. *sclerotiorum* were extensively proliferated in a subclade within clade 15 (see Fig. S1D). This proliferation of genes appeared to be primarily caused by the DNA transposon TcMar-Fot1 in both fungi (see Fig. S2A). Without these duplicated genes, *C*. *scovillei* only contained 36 homeodomain superfamily genes, similar to its sister species *C*. *nymphaeae* (see Fig. S1C). Unlike *HOX* genes and the homeodomain superfamily sparsely scattered in the genome of *C*. *scovillei*, these genes were located in clusters throughout the genome (see Fig. S2B). This phenomenon may be driven by the activity of a DNA transposon.

10.1128/mBio.01620-21.2 FIG S2 Phylogenetic tree of proliferated subclade genes and distribution of homeodomain superfamily and subcellular localization of CsHOX2 and CsHOX7 in *Colletotrichum scovillei*. (A) Phylogenetic tree containing proliferated genes that encode proteins with a homeobox-like domain from *C*. *scovillei* and Sclerotinia sclerotiorum. For each gene, repeat contents 1.5 kb upstream are represented as colored circles outside leaf nodes. Leaf nodes from *C*. *scovillei* are highlighted in light red, *S*. *sclerotiorum* in pink, and other *Colletotrichum* species in dark cyan. (B) Locations of homeodomain superfamily genes depicted using PhenoGram. Blue, green, and red circles represent homeobox genes, homeobox-like genes, and duplicated homeobox-like genes, respectively. Chromosomes (scaffolds) without any homeodomain genes are not shown. (C and D) Subcellular localization of CsHOX2 and CsHOX7 in developments of *Colletotrichum scovillei*. Conidial suspensions of representative transformants were placed on hydrophobic coverslips and detected by fluorescent microscope. The conidium of *C. scovillei* commonly possesses one nucleus. In response to the hydrophobic surface of coverslips, the first round of mitosis occurred within 2 h, followed by a septum formed in the middle of conidium. The germ tube emerged after appearance of septum and differentiated into an appressorium, which was accompanied with second round of mitosis within 4 h. In contrast to *M. oryzae*, nuclei in conidia of *C. scovillei* were never degenerated despite extending incubation time. Scale bar, 10 μm. (C) Subcellular localization of CsHOX2 during appressorium development. The CsHOX2:sGFP emerged and localized in nuclei of conidium before first round of mitosis, but disappeared after first round of mitosis (1.5 h). (D) Subcellular localization of CsHOX7 during appressorium development with or without 5 mM cAMP and 500 μM CaCl_2_ treatment. Treatment of cAMP and CaCl_2_ did not influence the localization of CsHOX7 during appressorium development. The CsHOX7:sGFP emerged and localized in nuclei of conidium after first round of mitosis (1.5 h) and in the nuclei of both the appressorium and conidium (8 h). Download FIG S2, TIF file, 2.3 MB. Copyright © 2021 Fu et al. 2021 Fu et al. https://creativecommons.org/licenses/by/4.0/ This content is distributed under the terms of the Creative Commons Attribution 4.0 International license.

As representatives of nuclear localization of HOX TFs in *C*. *scovillei*, we confirmed nuclear localization of CsHOX2 and CsHOX7 using transformants expressing the fusion proteins CsHOX2:sGFP and CsHOX7:sGFP, respectively, during fungal development (see Fig. S2C and S2D). HOX proteins of *C*. *scovillei* have been compared to known HOX proteins of *M*. *oryzae* and S. cerevisiae (33, 58). While S. cerevisiae has nine HOX protein-encoding genes, *M*. *oryzae* has seven; *C*. *scovillei* has three genes, in addition to the seven present in *M*. *oryzae* (see Fig. S2A). All HOX proteins in *C*. *scovillei* were found to be orthologous to those in *M*. *oryzae*, with the exceptions of CsHOX3, CsHOX8, CsHOX9, and CsHOX10. The homeobox domain in CsHOX8 with two C_2_H_2_ zinc finger domains and an HTH CenpB-type DNA-binding domain and CsHOX9 without any other domains were found to be paralogous to each other (see Fig. S2A). Only two *C*. *scovillei* proteins, CsHOX6 and CsHOX7, were found to be orthologous to the *HOX* genes in S. cerevisiae and *M*. *oryzae*.

### Generation of *CsHOX*-deletion mutants in *C*. *scovillei*.

To investigate the functional roles of *CsHOX* genes during fungal developments and infection of *C. scovillei*, we generated deletion mutants (*ΔCshox1* to *ΔCshox10*) for each *CsHOX* gene via homology-dependent targeted gene replacement. The deletion mutants were confirmed by Southern blotting and RT-PCR (see Fig. S3). Compared to the wild-type strain of *C*. *scovillei*, the deletion mutants displayed altered phenotypes during fungal developments and pathogenicity (Table 1; see also Fig. S4A to D). Briefly, the *ΔCshox2*, *ΔCshox3*, and *ΔCshox6* strains were reduced in conidiation; the *ΔCshox1*, *ΔCshox3*, *ΔCshox4*, and *ΔCshox5* strains produced abnormally morphological conidia; the *ΔCshox1* and *ΔCshox7* strains were impaired in appressorium formation in response to hydrophobic surface; the *ΔCshox1*, *ΔCshox7*, *ΔCshox8*, and *ΔCshox9* strains were defective in pathogenicity; the *ΔCshox2* strain caused normal anthracnose disease through appressorium-like structures developed from tips of mycelia. Moreover, the wild-type strain was not found to produce conidiomata, whereas the *ΔCshox1*, *ΔCshox3*, *ΔCshox5*, and *ΔCshox6* formed conidiomata on artificial medium (see Fig. S4B).

**TABLE 1:** Phenotypic characterization of *CsHOX* gene deletion mutants in *C*. *scovillei*^*a*^

Strain	Growth (mm)^*b*^		Conidiation (10^4^/ml)^*c*^	Conidial size (μm)^*d*^		Conidial germination (%)^*e*^	Appressorium formation (%)^*f*^
	CMA	MMA		Length	Width		
Wild-type	49.3 ± 0.3^A^	44.3 ± 0.5^ABC^	155.0 ± 9.1^A^	10.9 ± 1.3^D^	3.7 ± 0.5^B^	97.5 ± 1.3^A^	90.0 ± 3.2^A^
*ΔCshox1*	31.8 ± 0.5^E^	24.5 ± 0.9^H^	158.8 ± 12.9^A^	19.9 ± 2.4^A^	3.4 ± 0.3^C^	81.0 ± 4.4^D^	31.0 ± 6.7^B^
*Cshox1c*	48.5 ± 0.6^A^	44.0 ± 0.8^ABC^	148.5 ± 9.8^A^	10.6 ± 1.1^D^	3.8 ± 0.5^B^	97 ± 1.8^AB^	90.5 ± 4.5^A^
*ΔCshox2*	42.3 ± 0.5^D^	32.8 ± 0.9^G^	0^D^	ND	ND	ND	ND
*Cshox2c*	49.0 ± 0.8^A^	44.3 ± 1.3^ABC^	150.8 ± 6.4^A^	11.0 ± 1.2^D^	3.6 ± 0.3^B^	94.8 ± 2.2^ABC^	89.8 ± 5.1^A^
*ΔCshox3*	48.3 ± 0.3^A^	44.0 ± 0.8^ABC^	87.5 ± 6.5^C^	15.1 ± 2.3^B^	4.2 ± 0.3^A^	92.0 ± 3.2^BC^	88.8 ± 3.9^A^
*Cshox3c*	48.8 ± 0.9^A^	43.8 ± 0.5^ABCD^	153.0 ± 13.5^A^	10.9 ± 1.1^D^	3.7 ± 0.4^B^	96.8 ± 1.7^AB^	89.3 ± 5.5^A^
*ΔCshox4*	44.5 ± 0.6^C^	38.0 ± 0.8^F^	150.5 ± 8.8^A^	14.2 ± 1.2^C^	4.4 ± 0.4^A^	92.8 ± 2.2^ABC^	89.5 ± 4.4^A^
*Cshox4c*	48.8 ± 0.9^A^	44.8 ± 0.5^AB^	157.5 ± 13.0^A^	11.0 ± 1.6^D^	3.9 ± 0.4^B^	95.8 ± 2.1^ABC^	89.0 ± 4.0^A^
*ΔCshox5*	48.5 ± 0.6^A^	41.5 ± 0.6^E^	151.3 ± 7.0^A^	15.7 ± 1.0^B^	4.4 ± 0.3^A^	90.5 ± 2.6^C^	89.0 ± 5.0^A^
*Cshox5c*	48.5 ± 0.6^A^	44.5 ± 0.6^ABC^	146.8 ± 4.7^A^	10.9 ± 1.1^D^	3.6 ± 0.4^B^	95.3 ± 2.2^ABC^	88.8 ± 5.9^A^
*ΔCshox6*	47.3 ± 0.5^B^	43.3 ± 0.5 ^CD^	123.0 ± 6.3^B^	10.6 ± 1.2^D^	3.7 ± 0.4^B^	95.5 ± 3.4^ABC^	89.0 ± 5.4^A^
*Cshox6c*	48.8 ± 0.9^A^	44.3 ± 0.5^ABC^	148.5 ± 6.0^A^	10.9 ± 1.4^D^	3.7 ± 0.4^B^	94.8 ± 2.5^ABC^	90.0 ± 5.4^A^
*ΔCshox7*	48.5 ± 0.6^A^	42.5 ± 0.6^DE^	156.8 ± 15.9^A^	10.8 ± 1.3^D^	3.8 ± 0.3^B^	95.0 ± 2.2^ABC^	0^C^
*Cshox7c*	48.8 ± 0.7^A^	43.8 ± 0.9^ABCD^	150.0 ± 4.4^A^	10.9 ± 1.6^D^	3.8 ± 0.4^B^	94.5 ± 3.4^ABC^	89.3 ± 5.1^A^
*ΔCshox8*	48.0 ± 0.8^AB^	42.3 ± 1.3^BCD^	158.1 ± 9.8^A^	11.0 ± 1.3^D^	3.8 ± 0.3^B^	95.8 ± 2.2^ABC^	85.3 ± 4.3^AD^
*Cshox8c*	48.8 ± 0.9^A^	44.3 ± 0.9^ABC^	154.3 ± 19.6^A^	11.0 ± 1.2^D^	3.6 ± 0.4^B^	95.5 ± 2.1^ABC^	90.3 ± 3.5^A^
*ΔCshox9*	48.0 ± 0.8^AB^	43.3 ± 0.5 ^CD^	150.0 ± 7.7^A^	11.0 ± 1.4^D^	3.8 ± 0.3^B^	95.3 ± 2.5^ABC^	86.0 ± 3.7^AD^
*Cshox9c*	48.8 ± 0.8^AB^	45.0 ± 1.4^A^	155.5 ± 14.8^A^	11.0 ± 1.4^D^	3.7 ± 0.4^B^	94.5 ± 2.4^ABC^	88.8 ± 6.2^A^
*ΔCshox10*	48.8 ± 0.9^A^	43.8 ± 0.9^ABCD^	141.8 ± 12.6^A^	10.8 ± 1.3^D^	3.7 ± 0.3^B^	95.0 ± 2.2^ABC^	89.8 ± 4.6^A^
*Cshox10c*	49.0 ± 0.8^A^	43.8 ± 0.9^ABCD^	148.8 ± 9.4^A^	11.1 ± 1.3^D^	3.7 ± 0.3^B^	94.5 ± 2.9^ABC^	89.8 ± 5.0^A^

a Data are presented as means ± the standard deviations of three independent experiments, with three replicates per experiment. Significant differences were estimated using Duncan’s test (*P* < 0.05). The same superscript capital letters in a column indicate no significant difference. ND, not determined.

b Mycelial growth was measured at 6 days postinoculation on CMA and MMA.

c Conidiation was evaluated by counting the number of conidia collected with 5 ml of distilled water from 6-day-old V8 agar medium.

d Conidial size was determined by measuring the lengths and widths of at least 100 conidia.

e Percentage of conidial germination was measured at 12 h postinoculation on a hydrophobic surface using conidia from 6-day-old oatmeal agar. In each replicate, at least 100 conidia were measured.

f The percentage of appressorium formation was measured at 16 h postinoculation on a hydrophobic surface using conidia from 6-day-old oatmeal agar. In each replicate, at least 100 conidia were measured.

10.1128/mBio.01620-21.3 FIG S3 Targeted deletion of *CsHOX* genes in *Colletotrichum scovillei*. (A) Schematic representation of gene deletion. The gene deletion was performed with using an HPH cassette to replace each *CsHOX* gene based on homologous replacement. (B) Verification of gene deletion. The targeted deletion mutants for each *CsHOX* gene were confirmed by Southern blotting. (C) Verification of complementation. Transcripts of each *CsHOX* gene were detected in the wild type and the complemented strain, while they were not detected in deletion mutants. Download FIG S3, TIF file, 2.9 MB. Copyright © 2021 Fu et al. 2021 Fu et al. https://creativecommons.org/licenses/by/4.0/ This content is distributed under the terms of the Creative Commons Attribution 4.0 International license.

10.1128/mBio.01620-21.4 FIG S4 Phenotypic characterization of *CsHOX* deletion mutants. (A) Pathogenicity assays. Drops (20 μl) of conidial suspension (10^6^ ml^−1^) were placed on healthy unwounded and wounded pepper fruits and incubated in a humid plastic box at 25°C. The photographs of unwounded pepper fruits and wounded pepper fruits were taken after 9 and 6 days, respectively. Quantitative analysis of lesion area was performed by using ImageJ. The lesion size was normalized to the lesion caused by the wild type on unwounded pepper fruits as a relative of 1. (B) Roles of *CsHOX1*, *CsHOX3*, *CsHOX5*, and *CsHOX6* in conidiomata development on artificial medium. Mycelial agar plugs from 3-day-old MMA were inoculated into OMA and incubated with light for 20 days. (C) Appressorium formation on hydrophobic surface. Conidial suspensions (5 × 10^4^ ml^−1^) of the indicated strains were dropped on hydrophobic coverslips and incubated in a humid plastic box at 25°C. The photographs were taken after 16 h. (D) Pathogenicity assay of the *ΔChox2* strain. Three-day-old mycelial agar plugs (5 mm) were inoculated healthy unwounded pepper fruits and incubated in a humid plastic box at 25°C. The melanized appressorium-like structures were observed through a microscope after 3 days, and the photographs of anthracnose disease were taken after 12 days. Scale bar, 20 μm. Download FIG S4, TIF file, 1.8 MB. Copyright © 2021 Fu et al. 2021 Fu et al. https://creativecommons.org/licenses/by/4.0/ This content is distributed under the terms of the Creative Commons Attribution 4.0 International license.

### *CsHOX* genes are important for conidium production.

**FIG 1:**
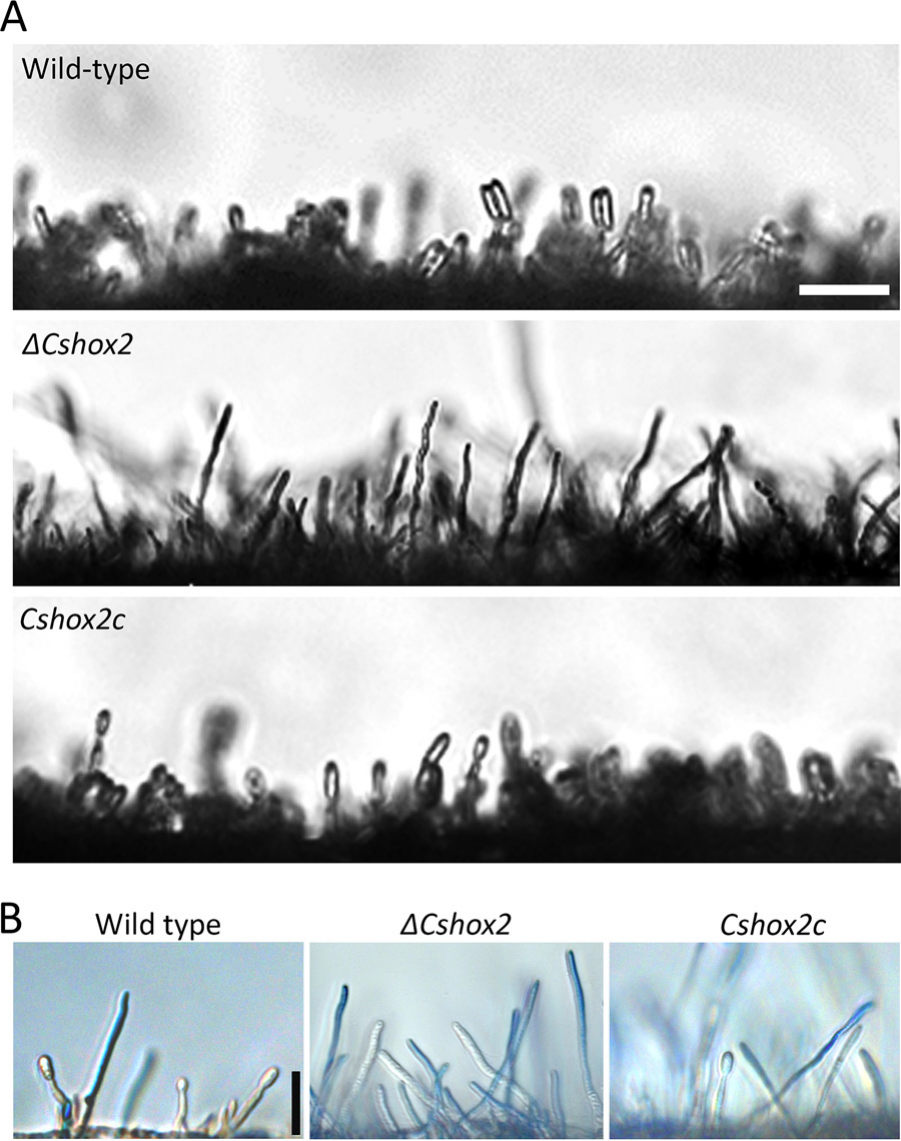
*CsHOX2* is essential for conidium production. (A) Production of conidia from conidiophores under inductive conditions. Photographs were taken after incubation of 3-day-old mycelial agar plugs in a humid box with light for 5 h. Scale bar, 25 μm. (B) Conidiophore development under inductive conditions. Aerial hyphae (blue) were stained by lactophenol aniline blue; conidiophores were not stained. Scale bar, 25 μm.

Anthracnose disease of pepper by *C*. *scovillei* is quickly spread via conidia in favorable environmental conditions. Therefore, we evaluated the conidiation of *CsHOX* gene deletion mutants. Quantitative analysis of conidia showed that *ΔCshox3* and *ΔCshox6* were significantly defective in conidiation, compared to the wild-type and complemented strains (Table 1). Notably, *ΔCshox2* was completely defective in conidiation, indicating that *CsHOX2* is essential for conidiation in *C*. *scovillei.* Microscopic investigation revealed that no conidia were observed in *ΔCshox2* with prolonged incubation, while the wild-type and *Cshox2c* strains developed dense conidiophores bearing conidia in 5 h (Fig. 1A). To further determine whether *CsHOX2* is involved in conidiophore formation, lactophenol aniline blue was applied to distinguish conidiophores from aerial hyphae. Microscopic examination indicated that wild-type, *ΔCshox2*, and *Cshox2c* strains developed conidiophores (Fig. 1B). Taken together, these results indicated that *CsHOX2* is a specific regulator of conidium production, and that *CsHOX3* and *CsHOX6* are quantitatively related to conidium production in *C*. *scovillei*.

### *CsHOX* genes are related to the morphology and germination of conidium, and stress response.

**FIG 2:**
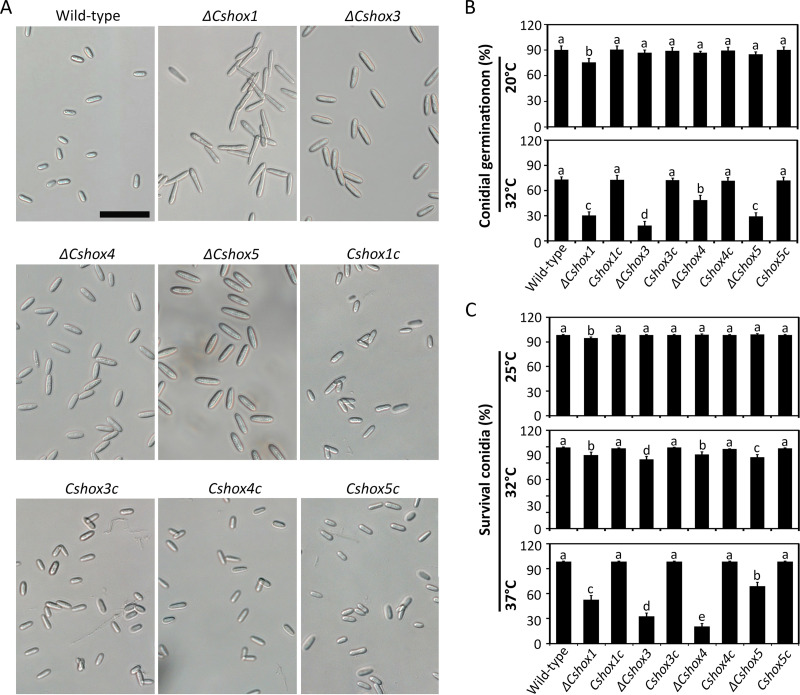
*CsHOX1*, *CsHOX3*, *CsHOX4*, and *CsHOX5* are important for the morphology and germination of conidia and for stress response. (A) Observation of conidial morphology. Conidia were collected with dH_2_O from 7-day-old oatmeal agar. Scale bar, 25 μm. (B) Effects of temperature on conidial germination. Conidial suspensions (5 × 10^4^ ml^−1^) were placed on hydrophobic coverslips and incubated in a humid box at 20°C and 32°C, which was controlled with respect to conidial germination at 25°C. (C) Effects of temperature on conidial longevities. Conidial suspensions (10^7^ ml^−1^) were placed at 25, 32, and 37°C for 16 h. Phloxine B (10 μg ml^−1^) was used to test percentage of survival conidia. At least 100 conidia were evaluated each time. Significant differences were estimated by Duncan’s test (*P* < 0.05). The same letter in a group with the same color indicates no significant difference.

Proper morphology and germination of conidium are prerequisites for efficient infection in conidium-mediated disease development. In terms of morphology, the *ΔCshox1*, *ΔCshox3*, *ΔCshox4*, and *ΔCshox5* mutants produced abnormal conidia, which were larger than the conidia of the wild-type and complemented strains (Fig. 2A and Table 1). This finding suggested that *CsHOX1*, *CsHOX3*, *CsHOX4*, and *CsHOX5* are important for conidial morphology. Conidial germination rates of the *ΔCshox1*, *ΔCshox3*, and *ΔCshox5* strains were significantly reduced, indicating that *CsHOX1*, *CsHOX3*, and *CsHOX5* are involved both in conidial morphology and germination (Table 1). When fungicides (e.g., dimethomorph, oxolinic acid, carbendazim, and fludioxonil) were applied to conidial germination assays, conidial germination of the *ΔCshox1* and *ΔCshox4* strains was significantly reduced by stress with dimethomorph (1 or 0.1 ppm) and carbendazim (1 ppm), respectively, compared to the germination of the wild-type and corresponding complemented strains (see Fig. S5A). In addition, germ tubes and conidia of the *ΔCshox1* strain displayed abnormal swelling following treatment with the chitin synthase inhibitor nikkomycin Z, compared to the wild-type and *Cshox1c* strains (see Fig. S5B). Moreover, we tested whether thermal stress affected the germination of conidia with abnormal morphology. The conidia of *ΔCshox1*, *ΔCshox3*, *ΔCshox4*, and *ΔCshox5* exhibited germination rates that were similar to the rate of the wild-type strain at 20°C, whereas the demonstrated significant inhibition of conidial germination at 32°C (Fig. 2B; see also Fig. S5C). Thus, we tested whether the conidial longevities of the *ΔCshox1*, *ΔCshox3*, *ΔCshox4*, and *ΔCshox5* strains were reduced by heat shock. Phloxine B was used to assess conidial survival. After incubation for 16 h, the ratios of conidial survival of *ΔCshox1*, *ΔCshox3*, *ΔCshox4*, and *ΔCshox5* strains were significantly reduced as the temperature increased from 25°C to 32 or 37°C (Fig. 2C; see also Fig. S5D). These results suggested that *CsHOX1*, *CsHOX3*, *CsHOX4*, and *CsHOX5* are involved in conidial morphology, germination, and viability.

10.1128/mBio.01620-21.5 FIG S5 Roles of CsHOX1, CsHOX3, CsHOX4, and CsHOX5 in conidial germination following stress treatments. (A) conidial germination subjected to fungicide treatments. Conidial suspensions (5 × 10^4^ ml^−1^) of indicated strains were mixed with 1, 0.1, and 0.01 ppm of dimethomorph, oxolinic acid, carbendazim, and fludioxonil. A conidial suspension with fungicide was placed on hydrophobic coverslips and incubated at 25°C for 12 h. A significant difference (*) of inhibition rate was estimated by Duncan’s test (*P* < 0.05). (B) Association of *CsHOX1* with cell wall integrity of the conidium and conidial germ tube. Conidial suspension (5 × 10^4^ ml^−1^) was dropped on hydrophobic coverslips, treated with (+) and without (–) nikkomycin Z (1 mM), and incubated for 2.5 h. Scale bar, 5 μm. (C) Roles of *CsHOX1*, *CsHOX3*, *CsHOX4*, and *CsHOX5* in the germination of conidia subjected to heat shock. Conidial suspensions (5 × 10^4^ ml^−1^) of the indicated strains were placed on hydrophobic coverslips and incubated at 25 and 32°C. The photographs of conidial germination were taken after 12 h. Scale bar, 20 μm. (D) Viability of conidia subjected to heat shock. Conidial suspensions (1.5 × 10^7^ ml^−1^) of indicated strains were incubated at 25, 32, and 37°C for 16 h and then stained with phloxine B (10 mg ml^−1^). The dead conidia were stained by phloxine B and exhibited red color. Scale bar, 50 μm. Download FIG S5, TIF file, 1.5 MB. Copyright © 2021 Fu et al. 2021 Fu et al. https://creativecommons.org/licenses/by/4.0/ This content is distributed under the terms of the Creative Commons Attribution 4.0 International license.

### *CsHOX7* is required for appressorium development but not for invasive growth.

**FIG 3:**
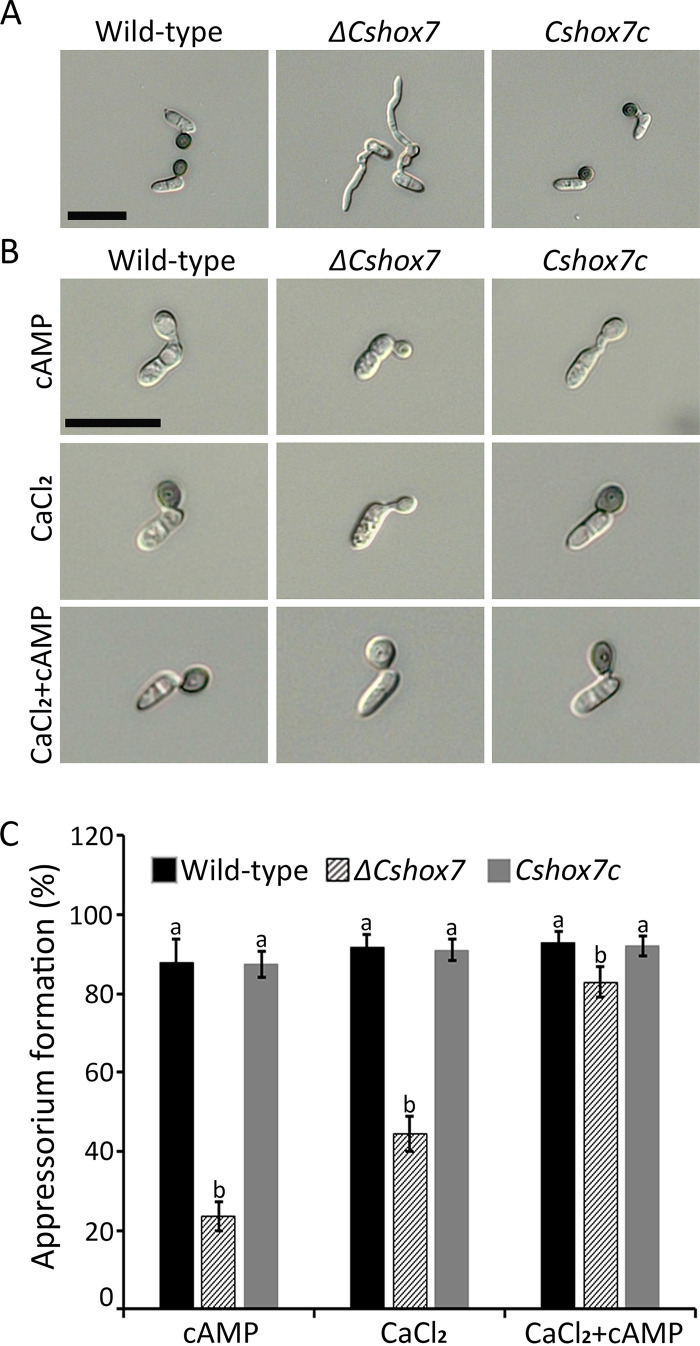
*CsHOX7* is important for appressorium formation on a hydrophobic surface. (A) Appressorium formation. Conidial suspensions (5 × 10^4^ ml^−1^) were placed on hydrophobic coverslips and incubated in a humid box at 25°C for 16 h. Scale bar, 20 μm. (B) Appressorium formation with exogenous 5 mM cAMP and 500 μM CaCl_2_ or with 5 mM cAMP and 500 μM CaCl_2_. Conidial suspension (5 × 10^4^ ml^−1^) was dropped onto hydrophobic coverslips and incubated in a humid box at 25°C for 16 h. CaCl_2_ and cAMP were added to the conidial suspension after 0 and 10 h, respectively. Scale bar, 20 μm. (C) Quantitative measurements of appressorium formation with addition of exogenous 5 mM cAMP and 500 μM CaCl_2_ or of 5 mM cAMP and 500 μM CaCl_2_. Appressorium formation rates were measured after 16 h on hydrophobic coverslips. Significant differences were estimated by Duncan’s test (*P* < 0.05). The same letter in a group indicates no significant difference.

Appressorium development is a key step in anthracnose disease development in *C*. *scovillei*. Strikingly, conidia of the *ΔCshox7* strain were completely abolished to produce appressoria, indicating that *CsHOX7* is essential for appressorium formation. A few swellings of the germ tube in the *ΔCshox7* strain indicated that the *ΔCshox7* strain retained the ability to sense the hydrophobic surface (Fig. 3A). Thus, we investigated the effects of exogenous chemical signals including cAMP, cutin monomers, and CaCl_2_ on the recovery of appressorium development in the *ΔCshox7* strain on hydrophobic coverslips. Conidia of the *ΔCshox7* strain were unable to form appressoria with the addition of exogenous cutin monomers. However, cAMP (5 mM) induced formation of 24% immature appressoria in the *ΔCshox7* strain, in contrast to 87.8% unmelanized appressoria in the wild-type strain (Fig. 3B and C). Compared to 91.5% formation of melanized appressoria by wild-type conidia, 44.5% of *ΔCshox7* conidia developed young appressoria with the addition of CaCl_2_ (0.5 mM) (Fig. 3B and C). These results revealed that exogenous cAMP or CaCl_2_ partially restored appressorium formation in the *ΔCshox7* strain. Treatment with both CaCl_2_ and cAMP increased the appressorium formation rate of the *ΔCshox7* strain to 82.8% and led to the formation of appressoria by 92.6% of wild-type conidia (Fig. 3C). Although appressoria of the *ΔCshox7* strain induced by treatment with cAMP and CaCl_2_ were not melanized, the sizes of appressoria were similar to those in the wild-type and *Cshox7c* strains (Fig. 3B). These compounds largely induced appressorium formation in the *ΔCshox7* strain, suggesting that *CsHOX7* is regulated by cAMP and Ca^2+^-dependent signaling pathways.

**FIG 4:**
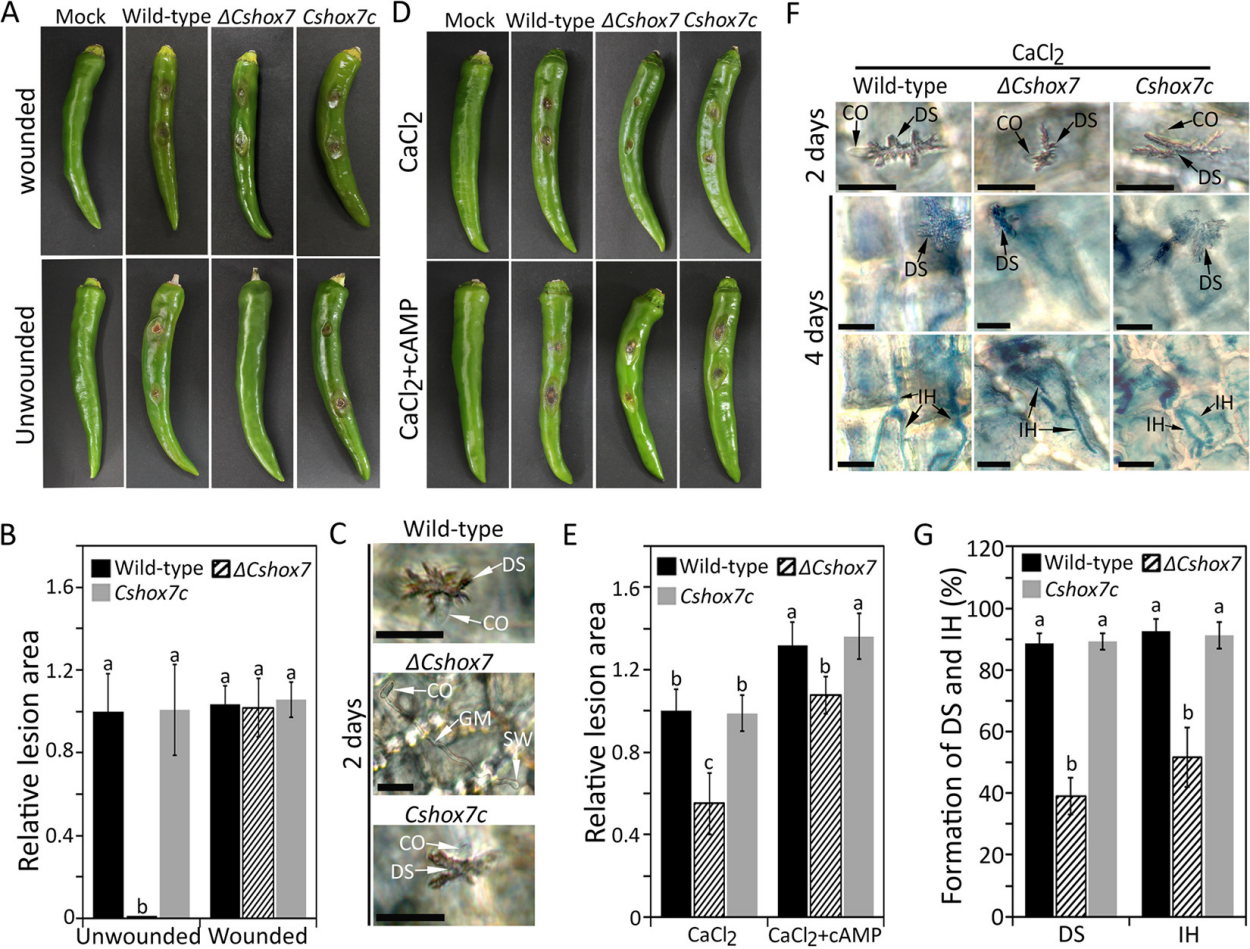
*CsHOX7* is required for appressorium-mediated penetration. (A) Conidium-mediated infection assay on pepper fruits. Healthy wounded and unwounded pepper fruits were inoculated with conidial suspensions (10^6^ ml^−1^) and placed in a humid box at 25°C. Photographs were taken after 6 days for wounded assays and after 9 days for unwounded assays. (B) Quantitative analysis of lesion area. The lesion area was measured by using ImageJ and normalized to the lesion caused by the wild-type in unwounded pepper fruit as a relative of 1. (C) Appressorium penetration on pepper fruits. Unwounded pepper fruits were inoculated with conidial suspensions (5 × 10^4^ ml^−1^) of indicated strains and placed in a humid box at 25°C for 2 days. Scale bar, 20 μm. (D) Recovery of conidium-mediated infection on pepper fruits. Unwounded pepper fruits were inoculated with conidial suspensions (10^6^ ml^−1^); 500 μM CaCl_2_ or 500 μM CaCl_2_/5 mM cAMP was then added. Photographs were taken after 9 days. (E) Quantitative analysis of relative lesion area. The lesion area was measured by using ImageJ and normalized to the lesion caused by the wild-type with exogenous addition of CaCl_2_ as a relative of 1. (F) Recovery of appressorium penetration on pepper fruits. Unwounded pepper fruits were inoculated with conidial suspensions (5 × 10^4^ ml^−1^) after the addition of 500 μM CaCl_2_. Dendroid structures and invasive hyphae (blue) were observed after 2 and 4 days, respectively. Microscopic images with differently focused layers of the same samples for visualization of dendroid structure (upper panel) and invasive hyphae (lower) are shown. Scale bar, 20 μm. (G) Quantitative analysis of dendroid structure formation and invasive hyphae growth. The percentage of dendroid structure (DS) formation was evaluated by examining at least 100 conidia and calculating the proportion of conidia that induced DS in host tissue. The percentage of invasive hyphae was evaluated by examining at least 100 DS and calculating the proportion of DS, from which the invasive hyphae grew into at least one adjacent host cell. CO, DS, and IH indicate the conidium, dendroid structure, and invasive hyphae, respectively. Significant differences were estimated by Duncan’s test (*P* < 0.05). The same letter in a group indicates no significant difference.

Compared to the wild-type strain, the *ΔCshox7* strain caused full infection of wounded pepper fruit but produced no lesions on unwounded pepper fruit, revealing that the *ΔCshox7* strain was unable to penetrate the host surface (Fig. 4A and B). Microscopic observation showed that conidia of the *ΔCshox7* strain formed swollen structures at the tips of germ tubes that failed to differentiate into appressoria on the host surface (Fig. 4C). Because exogenous addition of CaCl_2_ and cAMP partially restored appressorium formation in the *ΔCshox7* strain on hydrophobic coverslips (Fig. 3B and C), we tested whether exogenous CaCl_2_ and cAMP contributed to appressorium-mediated penetration of *ΔCshox7*. Exogenous cAMP failed to restore infection by the *ΔCshox7* strain (see Fig. S6A). However, conidia of the *ΔCshox7* strain successfully infected unwounded pepper fruits with the addition of exogenous CaCl_2_ (Fig. 4D and E). Treatment with both CaCl_2_ and cAMP facilitated the formation of lesions by the *ΔCshox7* strain on unwounded pepper fruits (Fig. 4D and E). Microscopic observation showed that the *ΔCshox7* strain was partially restored to form dendroid structure and grow invasive hyphae in unwounded pepper fruit with an addition of exogenous CaCl_2_ (Fig. 4F and G).

10.1128/mBio.01620-21.6 FIG S6 Roles of *CsHOX7* and *CsHOX1* in pathogenicity. (A) Recovery of appressorium-mediated penetration of the *ΔCshox7* strain on pepper fruits with the exogenous addition of cAMP. Unwounded pepper fruits were inoculated with conidial suspensions (10^6^ ml^−1^); 5 mM cAMP was then added after 12 h. Photographs were taken after 9 days. (B) Expression of *CsHOX1* during developments and infection. The expression of *CsHOX1* was evaluated by using qRT-PCR. Total RNA was extracted from different tissues, including mycelia, conidia, appressoria, and infected plants at 24, 48,72, and 96 h. MY, CO, AP, IF24, IF48, IF72, and IF96 indicate mycelia, conidia, appressoria, and infected plants at 24, 48, 72, and 96 h, respectively. (C) Detection of ROS in the first host cell after 3 days. Accumulation of H_2_O_2_ was observed only in the first pepper cell challenged by the *ΔCshox1* strain. Quantitative analysis of H_2_O_2_ accumulation. The H_2_O_2_ accumulation was estimated by examining at least 100 DSs and calculating the proportion of DS, which induced ROS accumulation (stained by DAB) in at least one adjacent host cell. Scale bar, 15 μm. (D) Gene Ontology (GO) classification of upregulated DEGs and whole genes from RNA-seq. The main GO categories include cellular component, molecular function, and biological process. Download FIG S6, TIF file, 1.6 MB. Copyright © 2021 Fu et al. 2021 Fu et al. https://creativecommons.org/licenses/by/4.0/ This content is distributed under the terms of the Creative Commons Attribution 4.0 International license.

### *CsHOX1* gene plays crucial roles in the development of anthracnose on pepper fruits.

**FIG 5:**
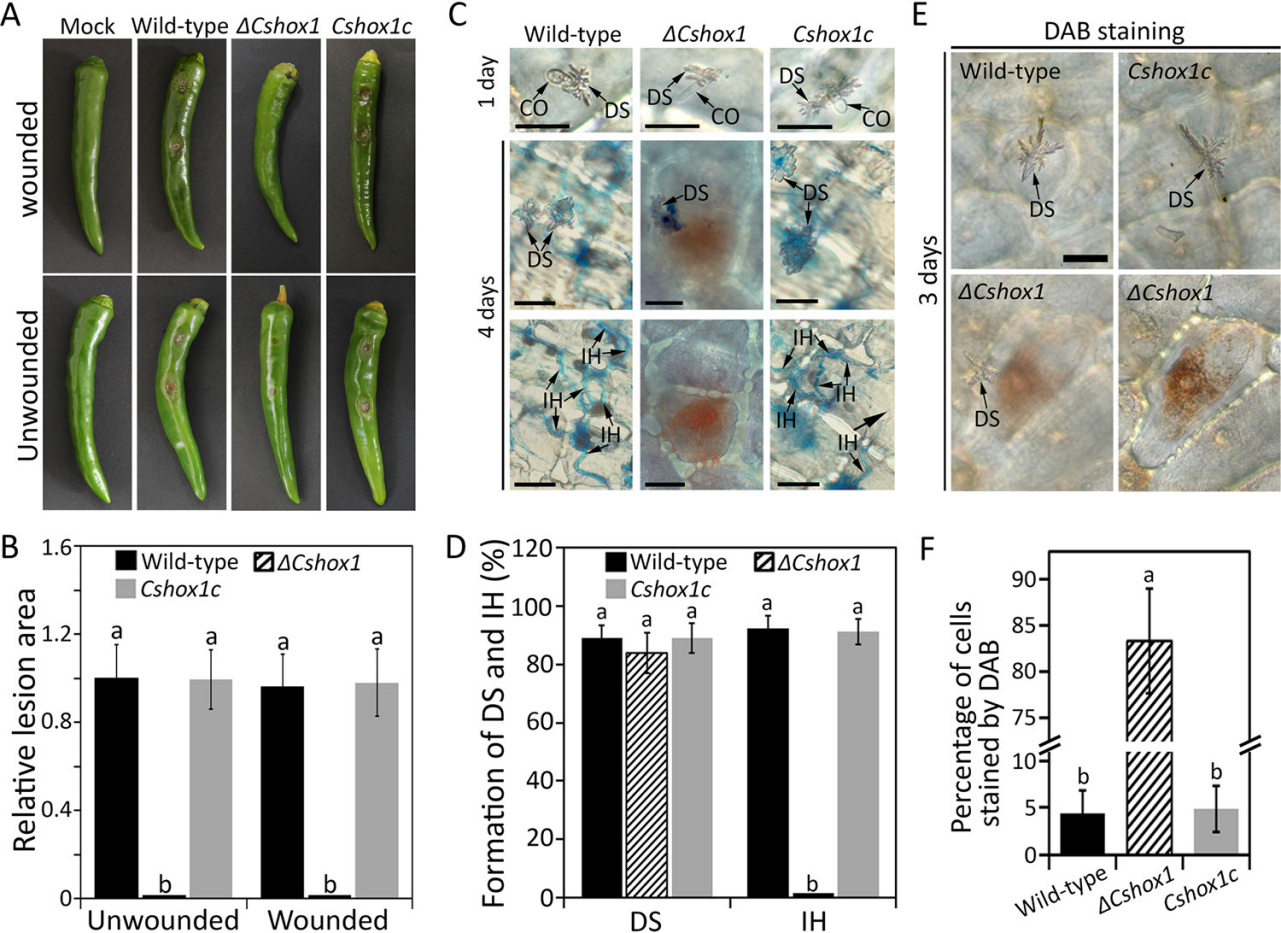
*CsHOX1* is required for invasive hyphae growth. (A) Conidium-mediated infection assay on pepper fruits. Healthy wounded and unwounded pepper fruits were inoculated with conidial suspensions (10^6^ ml^−1^) and placed in a humid box at 25°C. Photographs were taken after 6 days for wounded assays and after 9 days for unwounded assays. (B) Quantitative analysis of lesion area. The lesion area was measured by using ImageJ and was normalized to the lesion caused by the wild-type in unwounded pepper fruit as a relative of 1. (C) Appressorium penetration of on pepper fruits. Unwounded pepper fruits were inoculated with conidial suspensions (5 × 10^4^ ml^−1^) and placed in a humid box at 25°C. Dendroid structures were induced in the cuticle layer of pepper fruits after 1 day. Microscopic images with differently focused layers of the same samples for visualization of dendroid structure (upper panel) and invasive hyphae (lower) are shown. Scale bar, 20 μm. (D) Quantitative analysis of dendroid structure formation and invasive hyphae growth. The percentage of dendroid structure (DS) was evaluated by examining at least 100 conidia and calculating the proportion of conidia that induced DS in host tissue. The percentage of invasive hyphae was evaluated by examining at least 100 DSs and calculating the proportion of DSs, from which the invasive hyphae grew into at least one adjacent host cell. (E) Detection of ROS in the first host cell after 3 days. Accumulation of H_2_O_2_ was observed only in the first pepper cell challenged by the *ΔCshox1* strain. Scale bar, 20 μm. (F) Quantitative analysis of H_2_O_2_ accumulation. The H_2_O_2_ accumulation was estimated by examining at least 100 DSs and calculating the proportion of DS, which induced ROS accumulation (stained by DAB) in at least one adjacent host cell. CO, DS, and IH indicate conidium, dendroid structure, and invasive hyphae, respectively. Significant differences were estimated by Duncan’s test (*P* < 0.05). The same letter in a group indicates no significant difference.

The *ΔCshox1* strain failed to infect wounded pepper fruit and led to charcoal-colored spots without typical sunken lesions on unwounded pepper fruit, which is not a typical disease symptom (Fig. 5A and B). To figure out the roles of *CsHOX1* in development of anthracnose on pepper fruits, we performed microscopic observation and found that the *ΔCshox1* strain developed appressorium and penetrated the host surface, in a manner similar to that of wild-type and *Cshox1c* strains (Fig. 5C and D). Notably, in contrast to the wild-type and *Cshox1c* strains which grew invasive hyphae in host epidermal cells after 4 days, the host cells infected with the *ΔCshox1* strain exhibited no invasive growth (Fig. 5C and D). We further investigated the expression profile of *CsHOX1* with qRT-PCR and found that the *CsHOX1* gene was highly expressed in infection stage (see Fig. S6B). These findings suggested that *CsHOX1* is essential for invasive hypha development during anthracnose development on pepper fruits.

**FIG 6:**
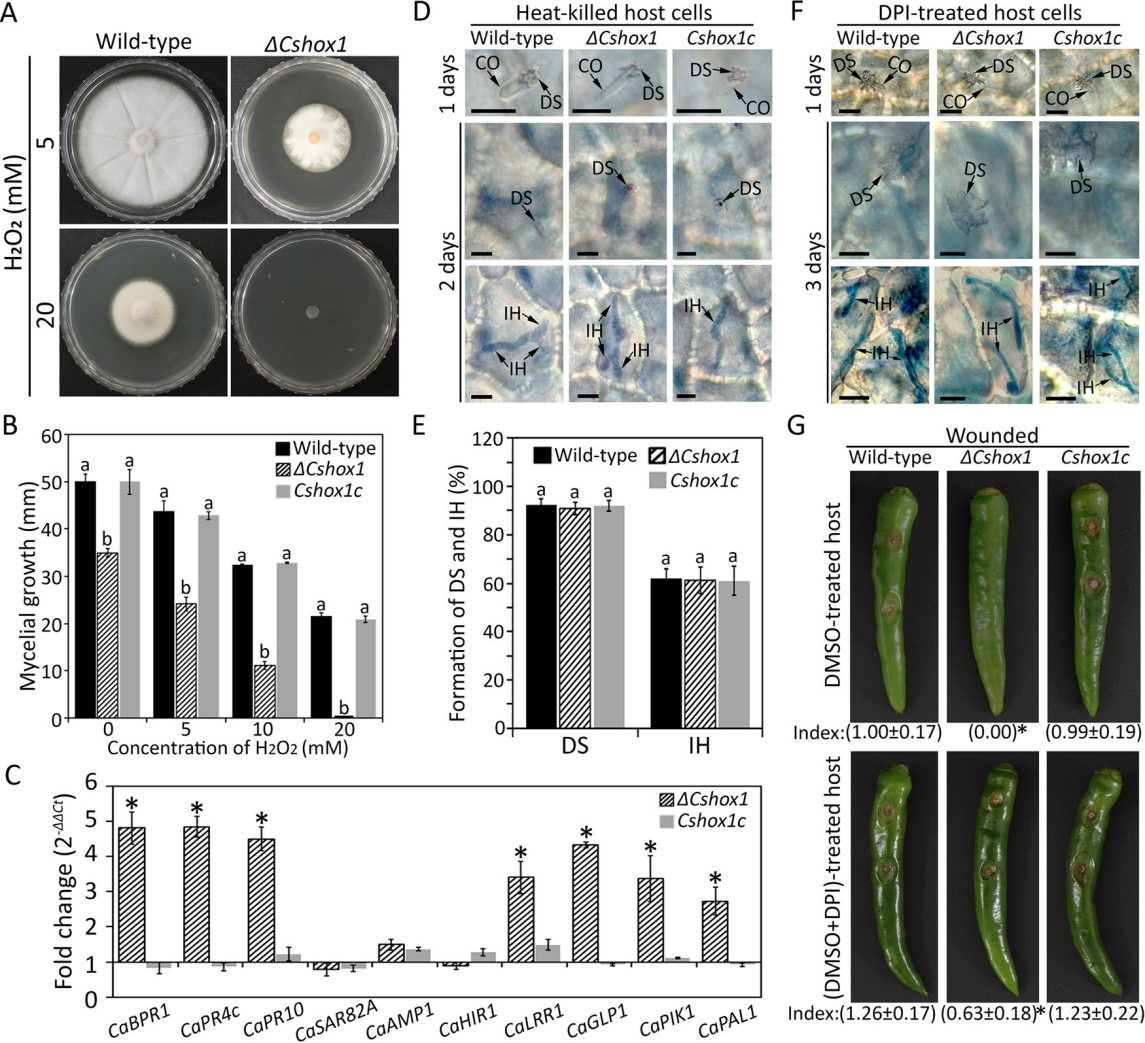
*CsHOX1* is involved in suppression of host resistance. (A) Photographs of mycelial growth on CMA containing 0, 5, 10, and 20 mM H_2_O_2_. The mycelia were grown without light for 6 days. (B) Quantitative analysis of mycelial growth. Growth rate of mycelia was evaluated by measurement of diameter of colony growth after 6 days. (C) Expression of host defense-related genes in pepper fruit tissues infected with the *ΔCshox1* strain, compared to tissues infected with the wild-type strain. qRT-PCR was performed, and the pepper actin gene was used as a reference. An asterisk (*) indicates a significant difference. (D) Observation of infection in heat-killed host tissues. The segments sliced from healthy pepper fruits were heated in boiling water for 30 s and then inoculated with conidial suspensions. The dendroid structure (DS) and invasive hyphae (IH) were observed at 1 and 3 days, respectively. Scale bar, 10 μm. (E) Quantitative analysis of DS and IH in heat-killed host tissues. The percentage of DS was evaluated by examining at least 100 conidia and calculating the proportion of conidia that induced DS in host tissue. The percentage of IH was evaluated by examining at least 100 DSs and calculating the proportion of DSs, from which the invasive hyphae grew into at least one adjacent host cell. (F) Observation of invasive growth in diphenyleneiodonium (DPI, suppressing ROS generation)-treated host tissues. The pepper fruits were first injected with DMSO containing DPI (2.5 μM) and then inoculated with conidial suspensions after 12 h. The dendroid structure and invasive hyphae were observed after 1 and 3 days, respectively. Scale bar, 10 μm. (G) Pathogenicity on DPI-treated pepper fruits. The wounded pepper fruits were drooped with 20 μl of DMSO (control) and 20 μl of DMSO containing DPI (2.5 μM) and inoculated with conidial suspensions after 12 h. Photographs were taken after 6 days. The index below each photo was the quantitative analysis of relative lesion area, and an asterisk (*) following the index of lesion area indicates a significant difference. The lesion area was measured by using ImageJ and was normalized to the lesion caused by the wild-type strain in DMSO-treated pepper fruits as a relative of 1. CO, DS, and IH indicate conidium, dendroid structure, and invasive hyphae, respectively. Significant differences were estimated by Duncan’s test (*P* < 0.05). The same letter in a group with the same color indicates no significant difference.

The host cells infected by the *ΔCshox1* strain exhibited brown pigmentation, which may reflect host cell defense response (59). Since host defense response is often accompanied by the rapid generation of reactive oxygen species (ROS), we performed the DAB staining experiment and found that pepper fruit cells infected with the *ΔCshox1* strain exhibited a high density of H_2_O_2_ accumulation in the first challenged epidermal cell, whereas host cells infected with wild-type and *Cshox1c* strains did not show accumulation of H_2_O_2_ (Fig. 5E and F; see also Fig. S6C). To test whether *CsHOX1* is involved in the degradation of ROS, we evaluated the mycelial growth rate on CMA containing H_2_O_2_. Mycelial growth of the *ΔCshox1* strain was significantly inhibited by 5 and 10 mM H_2_O_2_, and the *ΔCshox1* strain was unable to grow on CMA containing 20 mM H_2_O_2_, whereas wild-type and *Cshox1c* strains could grow on this artificial media (Fig. 6A and B), suggesting that *CsHOX1* is required for degrading of ROS. We further evaluated the expression of host defense-related genes and found that *CaBPR1*, *CaPR4c*, *CaPR10*, *CaHIR1*, *CaGLP1*, *CaPIK1*, and *CaPAL1* were significantly upregulated in pepper fruits infected by the *ΔCshox1* strain, compared to those infected by wild-type or *Cshox1c* strains (Fig. 6C; see also Table S2). This result indicated that CsHOX1 may be involved in suppression of host defense. Consistent with this hypothesis, we investigated the infection of the *ΔCshox1* strain in defense-compromised host plant by inoculating conidial suspensions onto diphenyleneiodonium (DPI; suppressing ROS generation)-treated and heat-killed pepper fruits. The results showed that the invasive growth of the *ΔCshox1* strain was comparable to that of wild-type and *Cshox1c* strains in heat-killed pepper fruits, and the infection of the *ΔCshox1* strain was partially rescued in in DPI-treated pepper fruits (Fig. 6D to G). Taken together, these findings suggested that *CsHOX1* is involved in the degrading of ROS and the suppression of host defense.

10.1128/mBio.01620-21.8 TABLE S2 Summary of functions of selected host defense-related genes in chili pepper. Download Table S2, XLSX file, 0.01 MB. Copyright © 2021 Fu et al. 2021 Fu et al. https://creativecommons.org/licenses/by/4.0/ This content is distributed under the terms of the Creative Commons Attribution 4.0 International license.

### *CsHOX1* regulates the expression of putative virulence-related genes.

**FIG 7:**
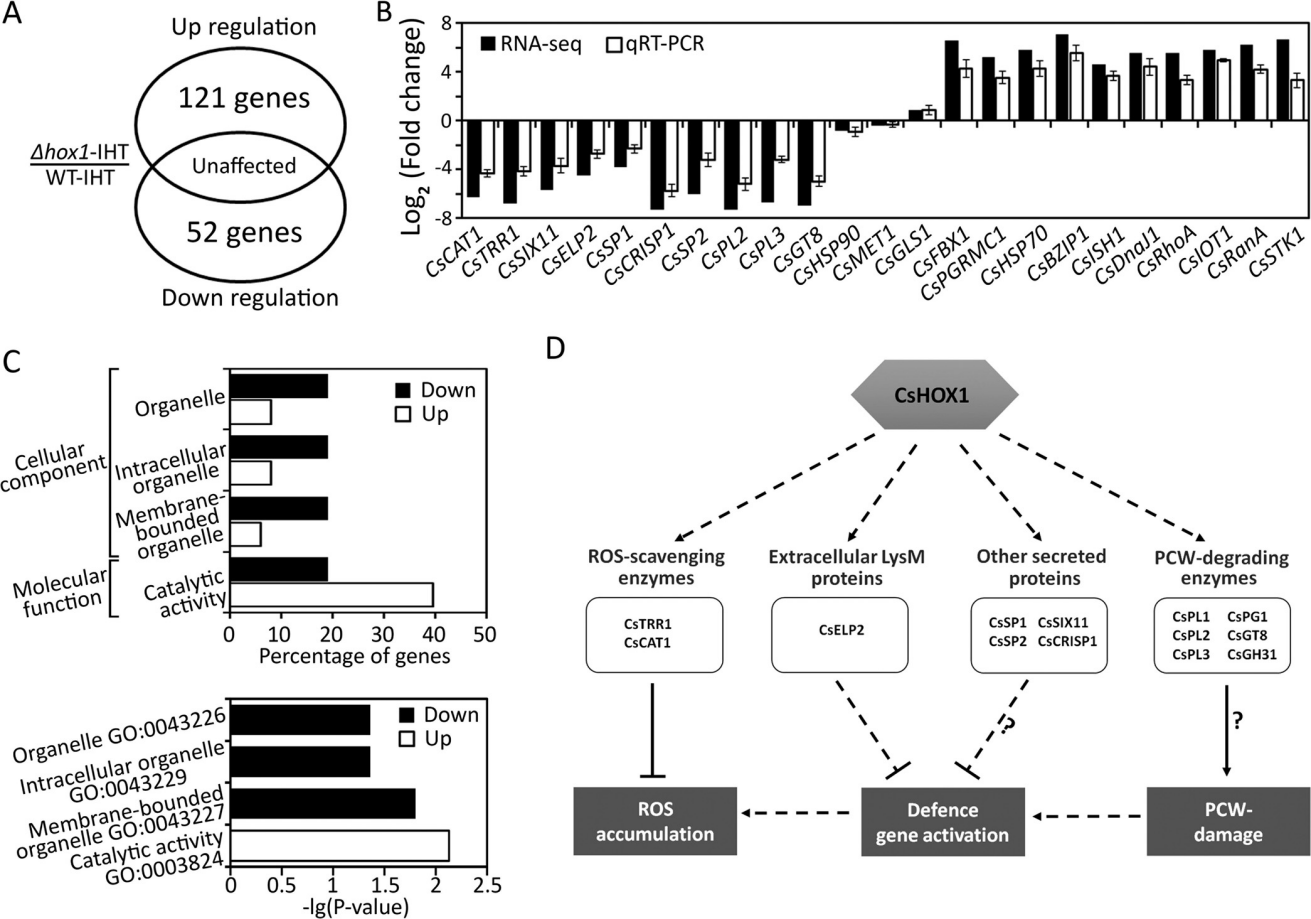
Analysis of CsHOX1-dependent genes and hypothetical model of pathogenicity-related genes regulated by CsHOX1 in *C. scovillei*. (A) Venn diagram showing the number of differentially expressed genes (DEGs) from *ΔCshox1* strain-infecting host tissues (*ΔCshox1*-IHT) and wild-type strain-infecting host tissues (WT-IHT). The RNA-seq was performed with three biological replicates, and the DEGs were identified using the criterion with a false discovery rate of <0.05 and a log_2_(fold change) of >2. (B) Validation of RNA-seq results by using qRT-PCR. The selected genes for qRT-PCR included 10 upregulated, 10 downregulated, and 3 unaffected genes. The information of selected genes was shown in the Table S3B. (C) The Gene Ontology (GO) classification of upregulated and downregulated genes from RNA-seq. The main GO categories include cellular component and molecular function. (D) Hypothetical model of pathogenicity-related genes regulated by CsHOX1 in *C. scovillei*, based on the downregulated DEGs of *ΔCshox1* strain-infecting host tissues (*ΔCshox1*-IHT). Deletion of *CsHOX1* caused induction of host defense, including defense gene activation and ROS induction. Among the downregulated DEGs of *ΔCshox1*-infecting tissues, CsTRR1 and CsCAT1 encode ROS-scavenging enzymes. CsELP2 encodes an extracellular LysM protein, which by suppresses the chitin-triggered immunity. Moreover, the genes encoding plant cell wall (PCW)-degrading enzymes (CsPL1, CsPL2, CsPL3, CsPG1, CsGT8, and CsGH31), significantly downregulated in the CsHOX1 deletion mutant, may cause PCW damage, thus triggering host defense. The four secreted proteins (CsSP1, CsSP2, CsSIX11, and CsCRISP1) may suppress the host defense, which remains further investigation. The information of all genes in the hypothetical model was shown in the Table S3C.

To further investigate the *CsHOX1*-dependent genes during anthracnose development in pepper fruits, we performed the RNA-seq to profile the gene expression of host tissues infected by the *ΔCshox1* strain versus the wild-type strain. The result showed that 52 genes were significantly downregulated (log_2_-fold change, <−2; *P* < 0.05), and 121 genes were significantly upregulated (log_2_-fold change, >2; *P* < 0.05) in host tissues infected by the *ΔCshox1* strain in reference to the wild-type strain (Fig. 7A; see also Table S3A). To validate the RNA-seq result, we evaluated the 10 upregulated differentially expressed genes (DEGs), 10 downregulated DEGs, and 3 unaffected genes to evaluate their expression with qRT-PCR (Fig. 7B). The expression of 10 selected upregulated genes, encoding putative pathogenicity-related secreted proteins, ROS-scavenging enzymes, and plant cell wall-degrading enzymes (see Table S3B), was consistent with that obtained from RNA-seq. The transcripts of 10 selected downregulated genes, involved in stress response and signal transduction (see Table S3B), were also similar to that from RNA-seq. The Gene Ontology (GO) term enrichment test showed that none of the functions in downregulated genes enriched compared to the whole gene sets, but the upregulated genes were enriched with functions involved in many cellular components, bindings, and metabolic processes (see Fig. S6D). The enrichment test between down- and upregulated genes showed genes related to organelles in the cellular component category were enriched in the downregulated genes, and genes with catalytic activity were enriched in upregulated genes (Fig. 7C). This result indicates that *CsHOX1* may play an important role in regulation of genes associated with the organelle formation and the catalytic activities. Further analysis of downregulated genes revealed that 13 genes were putative pathogenicity factors, including two ROS-scavenging enzymes (60, 61), five secreted proteins (62), and six plant cell wall-degrading enzymes (see Table S3C) (63). Many genes were upregulated in the *ΔCshox1* strain, including regulators such as protein kinases, phosphatases, and transcription factors. The RNA-seq data supported that the *CsHOX1* plays a key role in regulating anthracnose development of *C. scovillei* on pepper fruits.

10.1128/mBio.01620-21.9 TABLE S3 (A) DEG analysis between wild-type and *ΔCshox1* strains infecting the chili peppers. (B) The genes were selected to valid RNA-seq result with qRT-PCR. (C) List of candidate genes related to fungal pathogenicity. Download Table S3, XLSX file, 0.04 MB. Copyright © 2021 Fu et al. 2021 Fu et al. https://creativecommons.org/licenses/by/4.0/ This content is distributed under the terms of the Creative Commons Attribution 4.0 International license.

## DISCUSSION

TFs play critical roles in development and pathogenicity in organisms, including fungal pathogens. Essential functions of the evolutionarily conserved homeobox TFs have been demonstrated in several eukaryotes (35, 64). In this study, analysis of TFomes and homeodomain superfamily genes in *C*. *scovillei* was extended to the *Colletotrichum* species complex and selected outgroup species. These analyses revealed that *C*. *scovillei* has a unique feature within the *Colletotrichum* species complex; notably, the fungus exhibited an extremely high number of homeodomain superfamily genes, while *HOX* genes were evenly distributed among the species complex (Fig. 1A). In terms of InterPro domain relationships, the homeobox-like domain superfamily (IPR009057) is one of the largest gene families in the kingdom Fungi, inclusive of the homeobox domain (IPR001356) in *HOX* genes (40). This duplication event was observed in both *C*. *scovillei* and *S*. *sclerotiorum*, an outgroup species (Fig. 1A); this shared feature was attributed to the DNA transposon TcMar-Fot1 (Fig. 2A). While the expansion and enrichment of DNA transposons in the genome of *S*. *sclerotiorum* have been reported (65), the relationships of DNA transposons with the duplication of homeodomain superfamily genes are first reported here for both *S*. *sclerotiorum* and *C*. *scovillei*. Unlike tandem clusters of *HOX* genes in animals, *HOX* genes were found to be scattered throughout the genomes of fungi, including *C*. *scovillei* (Fig. 2B) (66). However, these homeobox-like genes were revealed to be duplicated multicopy genes located in a tandem manner in the *C*. *scovillei* genome (Fig. 2B). This is likely evidence for the recent transposon activity that occurred in *C*. *scovillei* after divergence from *C*. *nymphaeae* (see Fig. S1A), which presumably occurred approximately 2 million years ago (57, 67). Because transposable elements are the major drivers of fungal genome evolution (68), the association of transposons with homeodomain superfamily genes impacts genome organization and may affect cellular processes related to fungal development and pathogenicity.

Molecular mechanisms that govern anthracnose disease caused by *Colletotrichum* species on fruits have not yet been characterized. A large number of conidia are reproduced from phialides of conidiophores in many *Colletotrichum* species (69, 70). However, the genetic basis of the conidiation process remains largely unknown in the *Colletotrichum* genus. We found that the deletion of *CsHOX2* caused a complete defect in the production of conidia in *C*. *scovillei*, whereas the *ΔCshox2* strain developed normal conidiophores (Fig. 3). Considering that *MoHOX2* (orthologous to *CsHOX2*) is essential for *M*. *oryzae* conidiation in a stage-specific manner (33), the development of phialides on top of conidiophores, which are not clearly distinguishable, may be normal in the *ΔCshox2* strain. However, *HTF1* genes in Fusarium graminearum, F. verticillioides, and F. oxysporum (all orthologous to *CsHOX2*) were revealed to be involved in the formation of clearly differentiated phialides (50). The *ΔFghtf1*, *ΔFvhtf1*, and *ΔFohtf1* strains alternatively produce macroconidia directly from hyphae at low frequencies, indicating functional differences in *CsHOX2* from orthologous *HTF1* genes in the three Fusarium species. Unlike the complete defect of the *ΔCshox2* strain in conidium production, it is notable that the deletions of *CsHOX1*, *CsHOX3*, *CsHOX4*, and *CsHOX5* resulted in the development of abnormally large conidia (Fig. 4A); in contrast, deletion of *MoHOX4* (orthologous to *CsHOX4*) resulted in the production of abnormally small conidia in *M*. *oryzae* (33). Notably, 4 of 10 *HOX* genes are associated with conidial size and morphology in *C*. *scovillei*. Several lines of evidence suggest that the four genes play distinct roles in conidium development of *C*. *scovillei*. For example, the *ΔCshox1* strain produced significantly larger conidia (19.9 ± 2.4 μm) compared to those from the *ΔCshox3*, *ΔCshox4*, and *ΔCshox5* strains (3.4 ± 0.3 μm, 15.0 ± 1.7 μm, and 4.2 ± 0.5 μm) (Table 1). Abnormal swelling of the conidia and germ tubes was observed in *ΔCshox1* alone (not in the *ΔCshox3*, *ΔCshox4*, and *ΔCshox5* strains), following treatment with nikkomycin Z; this indicated a difference in cell wall integrity (see Fig. S4B). The sensitivity of conidia in the *ΔCshox3* strain was much higher following treatment with the fungicide carbendazim, compared to the sensitivities of conidia in the *ΔCshox1*, *ΔCshox4*, and *ΔCshox5* strains (see Fig. S4A), which support functional differences among the four *CsHOX* genes in *C*. *scovillei.* The *CsHOX1* gene is a critical regulator of cell wall integrity and conidia morphology, as well as pathogenic development, in *C*. *scovillei* (Fig. 4A and 7E; see also Fig. S4B), which represents a novel function of homeobox TFs in plant-pathogenic fungi.

The *ΔCshox1* strain was not defective in appressorium formation and host penetration on pepper fruits (Fig. 5C and D). The invasive hyphae of the *ΔCshox1* strain were unable to grow in the epidermal cells of pepper, following successful penetration into the cuticle layer via appressoria (Fig. 5C). The accumulation of the brown cloud and ROS only in the first epidermal cell challenged by the *ΔCshox1* mutant (Fig. 5E and F), and the significant upregulation of host defense-related genes were indicative of the activation of innate host immunity (Fig. 6C; see also Table S2). The increased sensitivity of the *ΔCshox1* mutant to H_2_O_2_, compared to the wild-type and *Cshox1c* strains, indicates that *CsHOX1*-regulated genes may be related to the modulation of ROS that are encountered in host pepper cells during anthracnose development of *C*. *scovillei* (Fig. 6A and B). Notably, the ROS-scavenging enzymes CsTRR1 and CsCAT1, orthologous to *M. oryzae* TRR1 and Sclerotinia sclerotiorum SCAT1, respectively, were significantly downregulated in gene expression profiles (see Table S3C). Both TRR1 and SCAT1 were reported to play important roles in ROS detoxification and plant infection (60, 61). The significant downregulation of *CsTRR1* and *CsCAT1* in *ΔCshox1*-infecting host tissues revealed the involvement of *CsHOX1* in ROS detoxification. Moreover, an LysM protein CsELP2, orthologous to *C. higginsianum* ChELP2, were found in the significantly downregulated genes (Fig. 7B). The ChELP2 encodes an extracellular LysM protein, which suppresses chitin-triggered plant immunity (71). The fungal chitin is known to induce host ROS accumulation and defense gene activation (72). This suggests that the CsHOX1 may be involved in suppression of chitin triggered immunity through transcriptional regulation of CsELP2. When performing pathogenicity assay by using host defense-comprised tissues, the *ΔCshox1* could grow invasive hyphae as wild-type strain did on heat-killed host tissues (Fig. 6D and E), while the pathogenicity was partially restored on DPI-treated host (Fig. 6F and G). This result indicated that the CsHOX1-regulated genes may be involved in suppression of host defense. In addition, the other four genes encoding secreted proteins (CsSIX11, CsCRISP1, CsSP1, and CsSP2) were found to be significantly downregulated in *ΔCshox1* strain-infected host tissues (Fig. 7B). The CsSIX11 was predicted to be orthologs to a known effector, SIX11 (Secretion In Xylem) of Fusarium oxysporum, which was recently demonstrated to be dispensable for fungal virulence, while its role in host defense is still unknown (73). The *CsCRISP1* gene was predicted to encode a cysteine-rich secretory protein. Although the ortholog of CsCRISP1 was not characterized, the cysteine-rich secretory proteins were reported to be involved in suppression of plant immunity in Verticillium dahliae (74). The detailed mechanisms of CsSIX11 and CsCRISP1 await investigation. These findings suggested that CsHOX1 is an essential factor that contributes to anthracnose development by transcriptional regulation of genes involved in the modulation of ROS and host defense (Fig. 7D).

Our study of appressorium-mediated fruit disease development in *C*. *scovillei* demonstrated that it uses a different strategy, compared to other fungal pathogens, to effectively penetrate the thick cuticle layer (20 to 25 μm) of host cells. For example, *M*. *oryzae*, a model for foliar disease in rice, generates substantial turgor pressure inside the appressorium to directly reach invading epidermal cells through the host cell wall by means of a strong mechanical force (75). However, *C*. *scovillei* has evolved a highly sophisticated strategy in which the fungus achieves tiny pin-point entry via the appressorium and then grows in the thick cuticle layer with the formation of dendroid structures within the cuticle layer; it does not use a penetration peg (2 to 4 μm in length) that directly reaches the epidermal cells of the pepper fruit. Therefore, *C*. *scovillei* generates, possibly, lower turgor pressure inside the appressorium and maintains conidial viability after penetration, in contrast to the autophagic cell death of conidia that occurs following penetration of *M*. *oryzae* (76). This hypothesis is supported by the observation that unpigmented immature appressoria of *C*. *scovillei* are able to penetrate the cell wall of pepper fruit and cause anthracnose disease (data not shown).

In this study, we demonstrated the dynamics and proliferation of homeodomain superfamily TFs during the evolution of an ingroup of *Colletotrichum* species and outgroup members. Detailed functional analyses have revealed that members of the homeobox TF family in *C*. *scovillei* play a key role in fungal development and anthracnose disease on pepper fruit. Our study provides a fundamental basis for understanding the molecular mechanisms involved in conidiation, appressorium development, and anthracnose disease on fruits, which will contribute to the development of novel strategies for use in managing anthracnose disease on many economically important fruits.

## MATERIALS AND METHODS

### Data used in the study and prediction of TFomes and repeat elements.

The fungal genomes and proteomes used in this study were downloaded from the National Center for Biotechnology Information (NCBI) database (see Table S1A). TFs were identified from proteomes using InterProScan 5.35-74, based on previously reported fungal TFs (40). Repeat elements were annotated using RepeatMasker 4.0.9 with a fungal repeat library (20170127) from RepBase (77).

### Identification of genes influenced by *HOX* genes in *C*. *scovillei*.

*HOX* genes of *C*. *scovillei*, *M*. *oryzae*, and Saccharomyces cerevisiae and their domains were aligned using MAFFT 7.29 (78); their sequence identities were calculated using the sident program in the trimAl v1.2 package (79). The binding domains of yeast *HOX* genes (YHP1, YOX1, TOS8, and CUP9) were retrieved from the JASPAR2020 database and used to search potential binding sites in the genome of *C*. *scovillei* (80). The binding sites were identified by PWMScan using the repeat masked intergenic sequences of the genome (81). Genes within 2 kb downstream of binding sites were collected; level 3 Gene Ontology term functions were visualized using WEGO (20181101) (82).

### Fungal strains and culture conditions.

*C*. *scovillei* strain KC05, originally isolated from pepper (*C*. *annuum*) in Gangwon Province, South Korea (32), was used as the wild-type strain. The strain and its transformants were routinely grown on V8 agar medium (80 ml liter^−1^ V8 juice and 15 g liter^−1^ agar powder), potato dextrose agar medium (39 g liter^−1^ powder; Kisan Bio, Seoul, South Korea), or oatmeal agar (50 g liter^−1^ oatmeal and 15 g liter^−1^ agar powder) with fluorescent light at 25°C (83). Mycelia for DNA or RNA extraction were cultured in liquid complete medium (CM; 6 g liter^−1^ yeast extract, 6 g liter^−1^ Casamino Acids, and 10 g liter^−1^ sucrose) or on TB3 agar medium (200 g liter^−1^ sucrose, 10 g liter^−1^ glucose, 3 g liter^−1^ yeast extract, 3 g liter^−1^ Casamino Acids, and 8 g liter^−1^ agar powder).

### Localization of histone H1:DsRed and CsHOX:sGFP fusion protein.

The histone H1:DsRed and CsHOX:sGFP fusion vectors were constructed by overlap cloning method (84, 85). PCR product of histone H1 (1.1 kb) was amplified with paired primers CAP_006988_F/CAP_006988_R (see Table S4), from wild-type genomic DNA. Also, 5.6 kb of DsRed fragment containing *HPH* gene and promoter of Neurospora crassa isocitrate lyase (ICL) encoding gene was amplified with the primer pair pIG-006988_F/pIG-006988_R from pIGPAPA-DsRed (84). The histone H1 fragment and DsRed fragment were combined by using an overlap DNA cloning kit (Elpis Biotech, Daejeon, South Korea), in which DsRed is under the control of Neurospora crassa *ICL* promoter. The recombinant vector of histone H1:DsRed was transformed into wild-type protoplasts. *CsHOX* fragments containing 1.5 kb of the promoter region and ORF (open reading frame) minus stop codon of *CsHOX2* or *CsHOX7* genes was amplified with the paired primers CAP_006569_F/CAP_006569_R or CAP_005766_F/CAP_005766_R from wild-type genomic DNA. As well, 5.0 kb of sGFP fragments including a Geneticin resistance gene was amplified with the paired primers pIG-006569_F/pIG-006569_R or pIG-005766_F/pIG-005766_R from pIGPAPA-sGFP (84). The *CsHOX* fragment and sGFP fragment were combined as described above, in which sGFP is fused to *CsHOX* and under the control of native *CsHOX* promoter. Each recombinant construct of CsHOX:sGFP was then transformed into protoplasts of transformants expressing histone H1:DsRed.

10.1128/mBio.01620-21.10 TABLE S4 Primers used in this study. Download Table S4, XLSX file, 0.02 MB. Copyright © 2021 Fu et al. 2021 Fu et al. https://creativecommons.org/licenses/by/4.0/ This content is distributed under the terms of the Creative Commons Attribution 4.0 International license.

### Generation of deletion mutants and complemented transformants.

The targeted gene deletion vector was constructed based on a modified double-joint PCR (86). Briefly, the 1.5-kb region on upstream and downstream of target gene (*CsHOX1* to *CsHOX10*) was amplified with the paired primers 5F/5R and 3F/3R of each gene, respectively (see Table S4). The *hygromycin B phosphotransferase* (*HPH*) gene was amplified with the primers HPHF/HPHR and fused with 1.5-kb upstream and downstream regions of each *HOX* genes by rounds of fusion PCR (87, 88). The fused deletion construct was then amplified in a nested PCR with the paired primers NF/NR. The product from nested PCR was introduced into the wild-type protoplasts by a polyethylene glycol-mediated transformation method as previously described (32). All transformants were first selected by a screening PCR with the paired primers SF/SR and finally confirmed by Southern blotting and RT-PCR. For complementation, the paired primers NF/NR were used to amplify each *HOX* gene. The PCR product and a 1.5-kb Geneticin resistance cassette were then cointroduced into protoplasts of each deletion mutant for *CsHOX* genes. The transformants of complementation were verified by RT-PCR.

### Nucleic acid manipulation and gene expression.

Fungal genomic DNA was prepared according to a quick and safe extraction method for general screening PCR, or a standard extraction method for Southern blot analysis (89, 90). The 500-kb DNA segment used as the Southern blot probe was amplified with the paired primers PF/PR (see Table S4). The genomic DNA was digested with specific restriction enzyme and then probed with that 500-kb DNA segment, which was previously labeled with Biotin-High Prime (Roche, Indianapolis, IN). To perform qRT-PCR and RT-PCR, total RNA was isolated from frozen fungal tissues and infected plant tissues using an Easy-Spin Total RNA extraction kit (iNtRON Biotechnology, Seongnam, South Korea). The complementary DNA (cDNA) was reverse transcribed from 5 μg of total RNA using SuperScript III first-strand synthesis system for RT-PCR kit (Invitrogen, Carlsbad, CA). The qRT-PCR mixture (10 μl) contained 1 μl of cDNA template (25 ng/μl), 1 μl of forward primer qrtF, 1 μl of reverse primer qrtR, and 5 μl of real-time PCR 2× master mix (Elpis, Daejeon, South Korea). The qRT-PCR experiment was performed with two replicates in three independent experiments using the StepOne real-time PCR system (Applied Biosystems, Foster city, CA). The PCR was set as follows: 95°C for 3 min (1 cycle), followed by 95°C for 15 s, 58°C for 30 s, and 72°C for 30 s (40 cycles). To measure the relative transcript abundance, *C_T_* values were normalized to those of *β‐tubulin* of *C. scovillei* or *Actin* of *C. annuum*. The normalized fold change of the target gene expression was expressed as 2*^−ΔΔCT^*, where *− ΔΔC_T_* = *ΔC_T _*_control_ − *ΔC_T _*_tested condition_ (91).

### Phenotypic characterization of mutants.

Vegetative growth of mycelium was evaluated by measuring the diameters of colonies grown on complete medium agar (CMA; 10 g liter^−1^ sucrose, 6 g liter^−1^ yeast extract, 6 g liter^−1^ Casamino Acids, and 15 g liter^−1^ agar powder) or minimal medium agar (MMA; 0.1 ml liter^−1^ trace elements, 30 g liter^−1^ sucrose, 2 g liter^−1^ NaNO_3_, 1 g liter^−1^ KH_2_PO_4_, 0.5 g liter^−1^ KCl, 0.5 g liter^−1^ MgSO_4_·7H_2_O, and 15 g liter^−1^ agar powder). Conidiation was evaluated by counting conidia collected with 5 ml of distilled water from 7-day-old V8 agar. Lactophenol aniline blue solution was used to distinguish conidiophores from mycelia (33, 92). For conidial germination and appressorium formation, drops of conidial suspension (20 μl; 5 × 10^4^ ml^−1^) were placed on hydrophobic coverslips, then incubated in a humid box. The numbers of germinated conidia and conidia with appressoria were counted in a sample of 100 conidia. To evaluate germination of conidia subjected to chemical stress, the conidial suspension was placed on hydrophobic coverslips; the fungicides dimethomorph, oxolinic acid, carbendazim, and fludioxonil were added at concentrations of 1, 0.1, or 1 ppm. To test the germination of conidia subjected to temperature stress, the conidial suspension was placed on hydrophobic coverslips and incubated at 20, 25, or 30°C. The chitin synthase inhibitor nikkomycin Z was used to test the cell wall integrities of conidia and germ tubes. Dead conidia were stained and distinguished by phloxine B in a conidial viability assay. Exogenous CaCl_2_ or cAMP was used to promote appressorium development. Data were collected from three independent experiments with at least three replicates per experiment. Significant differences were analyzed by Duncan’s test (*P* < 0.05).

### Plant infection assays.

The wounded plant infection assay was performed by placing a conidial suspension (10^6^ ml^−1^) on wounded healthy pepper fruit, which were then incubated in a humid box for 9 days at 25°C. For the unwounded plant infection assay, the conidial suspension was placed on unwounded healthy pepper fruit, followed by incubation for 6 days at 25°C. To observe fungal invasive hyphae inside pepper cells, a modified trypan blue solution containing 0.05% trypan blue (wt/vol), 30% phenol B (vol/vol), and 30% lactic acid (vol/vol) was applied. Briefly, healthy pepper fruits were inoculated with conidial suspension (5 × 10^4^ ml^−1^) and incubated for 4 days. The thin sections of infected pepper fruits were cut with a razor blade and fixed in a solution containing 10% (vol/vol) acetic acid, 30% (vol/vol) chloroform, and 60% (vol/vol) methanol for at least 16 h. The fixed samples were subsequently rehydrated in ethanol solutions of decreasing concentrations (100% [vol/vol], 70% [vol/vol], and 50% [vol/vol]) for at least 3 h at each concentration. The samples were then stained in modified trypan blue solution and destained with 70% (vol/vol) ethanol. The stained samples were embedded in 30% (vol/vol) glycerol and observed through a microscope. 3,3′-Diaminobenzidine (DAB) was used to stain hydrogen peroxide (H_2_O_2_) in pepper fruit cells. The thin sections of infected pepper fruits were infiltrated with DAB solution (1 mg ml^−1^ [pH 7.5]) under vacuum and boiled in 96% (vol/vol) ethanol for 10 min (93). The accumulation of iron (Fe^3+^) in tissues of pepper fruits was indicated by Perls Prussian blue staining according to previously reported methods (94, 95).

### RNA-seq and differentially expressed gene analyses.

The healthy fruits (green color) of chili pepper (*Capsicum annuum*) inoculated with 20 μl of distilled water or a conidial suspension (25 × 10^4^ conidia/ml) of the wild-type or the *ΔCshox1* strain were put in humid plastic boxes and incubated at 25°C for 48 h. The thin segments containing the outer cuticle layer, and epidermal cells were sliced from the infected pepper fruits with a razor blade. Each segment obtained from pepper fruit tissues was observed through a light microscope and confirmed to be infected by the wild-type or *ΔCshox1* strain. The confirmed segments were subsequently immersed in the liquid nitrogen. Each RNA sample was extracted from 30 segments using the Easy-Spin total RNA extraction kit (Intron Biotechnology, Seongnam, South Korea) according to the manufacturer’s instructions. The three biological replicates of extracted RNA samples were then sequenced by Illumina Hiseq 2500 (NICEM, Seoul, South Korea). The quality of raw read data from each sample was checked with fastp v0.21.0, including adaptor trimming and low-quality read filtering (96). The trimmed read data were mapped to the reference genome of *C. scovillei* using HISAT2 v2.2.1 (97), and the read count of reference genes was performed using StringTie2 v2.1.5 (98). The DEG analysis between the wild-type and *ΔCshox1* strains was performed using DESeq2 (99).

### Data availability.

The transcriptome data of *C. scovillei* wild-type and *ΔCshox1* strains were submitted to the SRA database in NCBI under accession numbers SRR13957602 to SRR13957604 and SRR13957605 to SRR13957607, respectively.
